# Supplementary material for: Knowledge, Attitudes and Practices of Indian Immigrants in Australia towards Oral Cancer and Their Perceived Role of General Practitioners: A Cross-Sectional Study
Source: Int J Environ Res Public Health. 2022 Jul 14;19(14):8596. doi: 10.3390/ijerph19148596 (PMC9319446; doi:10.3390/ijerph19148596)
Supplement: Supplementary file 1 [file ijerph-19-08596-s001.zip › ijerph-1783685-supplementary.pdf]

Date: ___________

Oral cancer risk?

Awareness matters……

This study aims to assess oral cancer risk among Indians in Australia. In this survey we ask you questions to explore your knowledge, attitudes and practices in relation to oral cancer risk. We are interested in understanding your perspective regarding oral cancer. Findings from this study may be used to improve health promotion materials to raise awareness of the risks of oral cancer. Your participation will help us to plan future activities to prevent oral cancer among Indians in Australia. We would appreciate your support, by completing this questionnaire.

- The survey will take 10-15 minutes to complete.
- Your identity will not be disclosed in any reports arising from this study.
- All the information you give will be treated in the strictest confidence.
- By completing the questionnaire, you are providing implied consent to participate in this study.
- For further information, please contact:

Nidhi Saraswat (Chief Investigator and PhD student),
email: 19278243@student.westernsydney.edu.au

SECTION A: ORAL HEALTH STATUS

1. **How would you rate the condition of your teeth and mouth on a scale of 1-5? (where 1 is "excellent" and 5 is "poor")** *Please Tick* √.

1	2	3	4	5
Excellent	Very Good	Good	Fair	Poor

2**. Do you currently have any problems or concerns with your teeth, gums or mouth?**

❒ No ⟶ *<u>Go to section B</u>*

❒ Yes ⟶ If yes, what are your main problems/concerns (*Tick* ☑ all that apply)

Swelling or lumps anywhere in your mouth ❒

Areas of red or white patches in your mouth ❒

Areas of ulcers (sores) in your mouth ❒

Pain in any area of your mouth ❒

Trouble while eating/chewing, swallowing or speaking ❒

Loose teeth ❒

Sore throat ❒

Difficulty in opening the mouth ❒

Other problems (Please Specify): ______________________ ❒

____

3. **Have your mouth problems/concerns affected your health?** (*Tick* ☑ that apply)

❒Yes, always ❒Yes, sometimes ❒ Never ❒ Don't know

If yes, how has it affected your health? Please explain:

____

____

4. Have you received advice from a dentist for this problem/concern? (*Tick* ☑ all that apply)

❒ Yes ❒ No

If no, why have you not seen a dentist? (tick ☑ all that apply)

I am nervous or scared to go	❒
I didn't think of it	❒
It costs too much	❒
I am too busy	❒
I am self-treating with over the counter products.	❒
Other (Please State): ________________________________	❒

____

SECTION B: KNOWLEDGE ABOUT ORAL CANCER RISK

1. Have you heard about mouth (oral) cancer? *(Tick ☑ in box)*

❑ Yes

❑ No → Go to question 4

❑ Can't remember → Go to question 3

2. How have you heard/learnt about mouth (oral) cancer? (*Tick* ☑ all that apply)

❑ Friends/Family

❑ GP/Specialist

❑ Dentist

❑ Media (Television, radio, newspaper)

❑ Social Media (facebook, Instagram, WhatsApp)

❑ Internet (websites)

❑ others, please specify____

3. Please indicate whether you think the following causes mouth (oral) cancer? *(Tick ☑ all that apply)*

	Yes	No
Smoking		
Alcohol		
Vitamin Deficiency		
Not brushing the teeth or gums		
Viruses or other germs		
Chewing tobacco		
Chewing Betel nut / ‘supari’		
Chewing Betel quid/’Pan’		
Frequent biting of lining of the mouth		
Putting pencils in mouth		
None of above		
Don’t know		

4. For each of the following statements, indicate whether you think it is true or false?
(*Tick* ☑ all that apply)

**A check up for mouth (oral) cancer:**	**True**	**False**	**Don’t know**
a) Is painless	❑	❑	❑
b) Is a way of finding mouth (oral) cancer at an early stage	❑	❑	❑
c) Helps in treatment of oral cancer if detected early	❑	❑	❑
d) Can be done by a GP	❑	❑	❑
**The signs/symptoms of mouth (oral) cancer are:**	**True**	**False**	**Don’t know**
a) A white patch/discoloration in the mouth	❑	❑	❑
b) An ulcer (sore) that does not heal	❑	❑	❑
c) A painless ulcer (sore) in the mouth	❑	❑	❑
d) A red patch in the mouth	❑	❑	❑
e) A yellow patch in the mouth	❑	❑	❑
f) A lump or swelling in the mouth	❑	❑	❑
g) A sore throat	❑	❑	❑
h) Discomfort or pain in the mouth	❑	❑	❑
i) Bleeding gums	❑	❑	❑

SECTION C: ATTITUDES TOWARDS ORAL CANCER RISK

1.How would you describe the importance of the following activities to prevent mouth (oral) cancer?
For each statement, please Tick ☑ *one box that comes closest to the way you think.*

**Activity**	**Very important**	**Fairly important**	**Important**	**Slightly important**	**Not important**
Doing exercise regularly	❐	❐	❐	❐	❐
Eating a healthy diet (2 fruits & 5 vegetables per day)	❐	❐	❐	❐	❐
Brushing teeth twice a day	❐	❐	❐	❐	❐
Visit a dentist at least once a year	❐	❐	❐	❐	❐
Visit a doctor (G.P.) regularly	❐	❐	❐	❐	❐

2. For each of the following statements, indicate whether you think it is true or false? (*Tick* ☑ all that apply)

**You are more likely to get oral cancer if you:**	**True**	**False**
a) Smoke tobacco, cigars or pipe	❑	❑
b) Smoke hukkah (sheesha)	❑	❑
c) Chew tobacco	❑	❑
d) Drink alcohol heavily	❑	❑
e) Chew gutkha	❑	❑
f) Chew betel quid/'pan'	❑	❑
g) Chew betel nut/'supari'	❑	❑
h) If your family got it	❑	❑

3. Do you think people of Indian background are more at risk of mouth (oral) cancer than local Australian people?
(Tick ☑ *one box)*

❐ Yes ❐ No ❐ Don't know

4.Why do you think people use products like tobacco preparations/betel nut/cigarettes/alcohol?

❑ Peer pressure (pressure from friends)

❑ Social status

❑ To connect with people

❑ Leisure/lifestyle/enjoy it/relaxation

❑ Addiction

❑ Family member use it

❑ It is a cultural practice for some Indians

❑ others, please specify____

5. For a white/coloured patch in the mouth (that has lasted more than 3 weeks) , please indicate which (if any) healthcare professional you would visit about that symptom. (*Tick* ☑ that apply)

❑ Doctor

❑ Dentist

❑ Pharmacist

❑ Other, please specify: ______

❑ Would not visit healthcare professional

6. For an ulcer/sore in the mouth (that has lasted more than 3 weeks), please indicate which (if any) healthcare professional you would visit about that symptom. (*Tick* ☑ that apply)

❑ Doctor

❑ Dentist

❑ Pharmacist

❑ Other, please specify: ________

❑ Would not visit healthcare professional

6. Are you seeing health professionals (Doctor/GP) from your cultural background for your regular health check-ups in Australia?

❑ Yes, I am seeing health professional from similar background

❑ No, I don't ⟶ Go to section D

If yes, what is the reason behind this? (*Tick* ☑ that apply)

❑ More knowledge

❑ More experience

❑ Easy to communicate

❑ More economic/affordable

❑ Cultural similarity

❑ Other (Please state____

____)

❑ No specific reason

SECTION D: PRACTICES RELATED TO ORAL CANCER RISK

1. Do you consume drinks that contain alcohol? (*Tick* ☑ all that apply)
❑ No, I have never tried alcoholic drinks ⟶ Go to question 2

❑ No, but I used to and stopped ⟶ Go to question 1.1

❑ Yes, I drink alcohol

If yes, how often do you consume these drinks? (*Tick* ☑ that apply)
❑ Monthly or less

❑ 2-4 times a month

❑ 2-3 times a week

❑ 4 or more times a week

2. Do you smoke tobacco? [this includes cigarettes, cigars, pipe, or hukkah (sheesha)]
(*Tick* ☑ that apply)

❑ No, I have never smoked

❑ No, but I used to and stopped

❑ Yes, I smoke

If yes, how often do you smoke?
Please give details of smoking habit in the table below by filling a number in box;
(where *0-Never, 1-Past, 2-Occasionally, 3-Daily)*

Smoking type	Type of user (choose a number 0-3 from instruction above)	Frequency (Numbers per day)
Cigarettes/Bidi/cigar		

3. Do you chew any product such as tobacco, betel nut/'supari', betel quid/'pan'?
(*Tick* ☑ that apply)

❑ No, I have never chewed ⟶ Go to section F

❑ No, but I used to and stopped ⟶ Go to question 3.1

❑ Yes, I chew

If yes, how often do you chew these products?

Please give details in the table below by filling a number in box; where

0 = *Never chewed*
1 = *Past- means having stopped the habit for more than one year*

2 = Occasional - means uses the habit less than daily.
3 = Daily

Type of Products	Type of User (choose a number 0-3 from instruction above)	Frequency (numbers per day)
Tobacco		
Betel quid/Pan		
Betel nut /supari/guthka		
Other- Specify ____________		

3.1 Did any of the following factors play a role in you starting the habit of chewing such products? (*Tick* ☑ all that apply)
❑ Friends/Family

❑ Peer pressure

❑ Media (Television, radio, newspaper)

❑ Social Media (facebook, Instagram)

❑ Internet (websites)

❑ Stressful time period

❑ While visiting India on holiday/business

❑ others, please specify____

3.2 If you used to chew such products, did any of the following play a role in you stopping this habit? (*Tick* ☑ all that apply)
❑ Friends/Family

❑ G.P/Specialist

❑ Dentist

❑ Media (Television, radio, newspaper)

❑ Social Media (facebook, Instagram)

❑ Internet (websites)

❑ others, please specify____

❑ Not applicable

3.3 How do you get these products? (*Tick* ☑ all that apply)
❑ Asian grocery stores

❑ Friends/ relatives visiting from India

❑ Indian restaurants

❑ Temples/religious places

❑ others, please specify____

SECTION E: YOUR ACCESS TO HEALTH CARE

1. Have you visited a doctor (GP) or community health clinic or practice nurse in last 12 months? (*Tick* ☑ all that apply)

❑ No ❑ Yes

If no, when was your last visit to a doctor?

1-2 years ago	❑
2 - 5 year ago	❑
More than 5 years ago	❑
Don't remember	❑

2. Have you visited a dentist in last 12 months? (*Tick* ☑ all that apply)

❑ No ❑ Yes

If no, when was your last visit to a dentist?

1-2 years ago	❑
2 - 5 year ago	❑

More than 5 years ago ❒

Don't remember ❒

3. Have you received any information about 'oral cancer' during your visits to the doctor (GP) or practice nurse or dentist? (*Tick* ☑ all that apply)

❒ No
❒ Don't remember → *Go to question 5*

❒Yes

If yes,

3.1 What information have you received? (*Tick* ☑ all that apply)
- ❒ Instructions about how to look after your mouth and teeth.
- ❒ Oral cancer risk information through educational material such as leaflets, pamphlets, samples or other.
- ❒ Other advice (please specify): ____

3.2 Where have you received this information from? (*Tick* ☑ all that apply)
❒Dentist ❒Doctor (G.P.) ❒ Practice nurse ❒ Other (Please state:_____________)

4. How satisfied are you with the information you received about 'oral cancer', on a scale of 1 to 5 (where 1 = "very satisfied" and 5 is "very dissatisfied")?
Please Tick √.

1	2	3	4	5

Very satisfied Very dissat isfied

Was the information easy to understand?

❒Yes ❒No

5. For each of the following statements, (*Tick* ☑ all that apply).

a)	Do you think doctor (GP) could assist you in identifying oral health problems like oral cancer?	❒Yes	❒ No	❒ Don't know
b)	Do you think doctors (GP) have sufficient knowledge about oral cancer to advise you?	❒Yes	❒ No	❒ Don't know

c)	Would you consider oral health advice given by your doctor (GP)?	❒Yes	❒ No	❒ Don't know
d)	Would you make an appointment to see a dentist if you were provided a dental referral by a doctor (GP)?	❒Yes	❒ No	❒ Don't know

SECTION F: ABOUT YOU

1. Personal Details:

Gender/sex:	
Age at your last birthday:	___________years
Were you born in Australia? (*Tick* ☑ that apply)	❒ Yes ❒ No<br>If yes, were your parents born in India?<br>❒ Yes ❒ No
How long did you live in India? (*Tick* ☑ that apply)	❒ Never lived ❒ lived for: ___years<br>❒ Don't remember
Do you visit India? (*Tick* ☑ that apply)	❒ Yes ❒ No
Area of Residence in Australia:	Suburb/Postcode:
Years since living in Australia:	
Language spoken at home:	
Marital status:	❒ Single ❒ Married/partnered<br>❒ Divorced ❒ Widowed

2. What do you consider as your current religion, if any?

❒ Prefer not to answer

3. Level of education

What is the highest level of educational you completed? *Tick* ☑ *one box*

Primary school	❒
High school	❒
TAFE	❒
University	❒
No formal education	❒

4. Employment Status (Please describe your work status by Ticking ☑ *one box)*

❒ Full time	❒ Part time	❒ Casual
❒ Not working	❒ Home/Domestic duties	❒ Retired

5. What is your average annual combined household income? *Tick* ☑ *one box*

Less than $40,000	❒
$40,000 to $59,000	❒
$60,000 to $79,000	❒
$80,000 to $99,000	❒
$100,000 to $120,000	❒
More than $120,000	❒
Don't know	❒
Prefer not to answer	❒

6. Do you have private health insurance that includes dental cover? *Tick* ☑ *one box*

❒ Yes ❒ No ❒ Don't know

7. Do you currently have the following cards? *Tick* ☑ all that apply**.**

Pensioner concession card	❒ Yes	❒ No	❒ Don't know
Health care card	❒ Yes	❒ No	❒ Don't know
Department of Veterans Affairs card	❒ Yes	❒ No	❒ Don't know

8. Do you have a family history of Cancer/mouth (oral) cancer? *Tick* ☑ *one box*

❒ Yes ❒ No ❒ Don't know ❒ Prefer not to answer

THANK YOU FOR YOUR TIME AND YOUR VALUABLE CONTRIBUTION TO THIS STUDY

If you would like to receive the summary of study results, please provide your contact details:

Phone: ____

OR Email id: ____

(Note: your information will remain anonymous)
